# Emotion regulation skills training for adolescents and parents in a clinical setting: a randomised controlled trial

**DOI:** 10.1186/s13034-026-01098-1

**Published:** 2026-05-28

**Authors:** Kristina Holmqvist Larsson, Fredrik Falkenström, Gerhard Andersson, Helene Stern, Linnéa Bolmgren, Therese Engström, Viktoria Källström, Mikaela Nilsson, Emma Pettersson, Floor Seesing, Trine Öhman, Kalle Österman, Maria Zetterqvist

**Affiliations:** 1 Department of Child and Adolescent Psychiatry in Linköping, Region Östergötland, Linköping, Sweden; 2 Center for Social and Affective Neuroscience, Department of Biomedical and Clinical Sciences, Linköping University, Linköping, Sweden; 3 Department of Psychology, Linnaeus University, Växjö, Sweden; 4 Department of Behavioural Sciences and Learning, Linköping University, Linköping, Sweden; 5 Department of Biomedical and Clinical Sciences, Linköping University, Linköping, Sweden; 6 Department of Clinical Neuroscience, Karolinska Institute, Stockholm, Sweden; 7 Psykologhälsan, Linköping, Sweden; 8 Department of Child and Adolescent Psychiatry in Norrköping, Region Östergötland, Norrköping, Sweden

**Keywords:** Emotion regulation, Emotion dysregulation, Skills training, Adolescents, Treatment, RCT

## Abstract

**Background:**

Emotion dysregulation is a transdiagnostic construct associated with psychiatric symptomatology. Improving strategies to regulate emotions is an important focus in several psychological treatments. In the current study we investigated the effects of a brief seven-session emotion regulation skills training group for adolescents and parents in a randomised controlled trial in a naturalistic setting.

**Methods:**

This non-blinded, randomised study recruited participants from two child and adolescent psychiatric outpatient clinics during Mars 2019 to January 2023. A total of 118 adolescents, 14 to 17 years old, were assessed and randomised to either a seven-session emotion regulation skills training group or to a control group. The intervention was transdiagnostic and open to all patients with few exclusion criteria. Both groups received treatment as usual consisting of multidisciplinary child and adolescent psychiatric care. Self-reported emotion dysregulation measured with the Difficulties in Emotion Regulation Scale was compared between the two arms after the intervention. Secondary outcomes were alexithymia, emotional awareness, symptoms of depression and anxiety and quality of life.

**Results:**

Of 58 adolescents randomised to emotion regulation skills training group, 48 started the intervention and 36 completed it and were included in the final analysis. In the control group, 41 participants of the 60 randomised remained in the final analysis. Of the primary outcome measure Difficulties in Emotion Regulation Scale, only one subscale, Lack of Emotional Clarity, significantly decreased for the emotion regulation skills training group compared to the control group. There were no significant differences in overall emotion dysregulation. Significant reductions of Alexithymia, measured by Toronto Alexithymia Scale, were found in the intervention group compared to the control group. No other significant differences were found between groups in secondary outcomes. Exploratory within-group analyses pointed to a reduction in overall emotion dysregulation, indicating a further effect at three months post-intervention.

**Conclusions:**

The brief group-based emotion regulation skills training for adolescents and parents in child and adolescent psychiatric outpatient clinics, delivered adjunctive to treatment as usual, appears to improve certain aspects of emotion regulation, specifically emotional clarity and alexithymia. While it did not show effects on psychiatric symptoms or quality of life, explorative analyses indicate that it may reduce overall emotion dysregulation over time.

*Trial registration* NCT03900533.

**Supplementary Information:**

The online version contains supplementary material available at 10.1186/s13034-026-01098-1.

## Introduction

Emotions are an inherent part of human existence. Being able to manage our emotions is therefore central to our well-being. Emotions communicate important information that is necessary for our survival. Experiencing fear, anger or sadness, for example, prompts us to seek safety, defend ourselves, or grieve. However, identifying, understanding and accepting our emotions, especially negative emotions, can be challenging, and difficulties with emotion regulation (ER), or emotion dysregulation (ED), negatively impact quality of life [1, 2].

Most definitions of ER emphasise the conscious or unconscious process where humans use different strategies to influence the intensity, duration, and expression of emotions in relationship to a specific goal, or as a response to environmental demands [3, 4]. Gratz and Roemer’s definition [5] of ER has clinical relevance for the understanding and treatment of ED. In this definition, awareness and acceptance of emotions and the ability to modulate emotional responses are emphasised. Adaptive ER requires control over impulsive behaviours in order to act according to long-term goals or values, even in the presence of negative emotions. Furthermore, emotional clarity and emotional awareness are constructs that refer to the ability to identify, discriminate, describe and understand emotions [6–8]. Low emotional clarity is associated with a higher use of maladaptive ER strategies, such as rumination and expressive suppression [7]. The concepts of emotional clarity and emotional awareness are closely related to alexithymia, which is a condition characterized by difficulties identifying, describing, and expressing one’s emotions [9]. High levels of alexithymia seem to hinder effective ER [9] and there is an association between high levels of alexithymia and maladaptive ER strategies [10]. Alexithymia is associated with psychopathology and is identified as a transdiagnostic risk factor for affective disorders [11, 12].

ED can lead to impulsive and risk-taking behaviour and lacking strategies to regulate emotions is associated with several negative outcomes, such as internalising problems, somatic complaints, symptoms of anxiety and depression, and externalising problems, such as delinquent and aggressive behaviour [2, 13, 14]. Further examples are nonsuicidal self-injury (NSSI) and substance use [15, 16]. Consequently, adaptive ER strategies have become an important focus in several psychological treatments [17, 18].

Since risk-taking behaviours tend to increase during adolescence, and psychopathology and mental disorders often have their onset during this time period [19], it is important to develop effective treatments for this age group. Different examples of treatments with ER components that target adolescents are Dialectical Behavioural Therapy for Adolescents (DBT-A; [20]), typically delivered in 19 to 24 weeks, The Unified Protocol for the Treatment of Emotional Disorders in Adolescents (UP-A; [21]), ranging from 8 to 21 weeks, and 12-week Internet-Delivered Emotion Regulation Individual Therapy for Adolescents (IERITA; [22]). Acceptance and Commitment Therapy (ACT; [23]) has also been used in adolescents, see Petersen [24] for an overview. ER treatments for adolescents have been successful in reducing NSSI, suicidal ideation, symptoms of depression, anxiety and borderline personality disorder, for example [22, 25–28], and improvement in ER strategies has shown to be a mechanism of change [29–31].

Learning how to deal with emotions, especially negative emotions, is a developmental challenge for adolescents [32]. Research findings emphasise parents’ role in children’s and adolescents’ development of ER strategies [33, 34], both in positive and negative directions. Morris et al. [35], emphasise that parental socialisation of ER is particularly important during adolescence as this is a critical period for neurobiological development [36], which has a direct impact on ER abilities. Knowledge about parents’ role in the development of ER strategies [34, 35], together with findings on the benefits of parental involvement in psychological interventions for adolescents [37], speak for the potential benefit of including parents in treatment.

Taken together, this argues for the importance of intervening early to reduce ED and improve ER skills in adolescents with mental health problems. It is also of interest to examine whether this can be achieved in a brief format. Due to potential difficulties getting patients to complete extensive interventions in clinical settings, a five-session emotion regulation skills training (ERST) group was evaluated in a feasibility study [38]. The ERST group was feasible with acceptable dropout rates and had preliminary promising results with decreased ED. Following the findings that five sessions were feasible, the ERST group was extended to seven sessions to increase the opportunity for participants to practice and generalize the content, although still being brief.

In the current study we report the results from a randomised controlled trial of a brief, transdiagnostic, ERST group focusing on reducing ED. The ERST group was delivered jointly to adolescents and parents in naturalistic CAP outpatient settings. The aim was to evaluate if the ERST group is an efficacious treatment. More specifically, we first wanted to examine whether adolescents in the intervention group who received ERST group + treatment as usual (TAU) reported lower levels of ED when compared to a control group consisting of only TAU. Secondly, we examined if adolescents receiving ERST group reported more emotional awareness, lower levels of alexithymia, lower levels of symptoms of depression and anxiety, and higher quality of life compared to controls. We hypothesised that the ERST group would result in reduced ED, alexithymia, depressive and anxiety symptoms and improved emotional awareness and quality of life.

## Method

### Study design

This was a non-blinded, randomised controlled intervention study in a naturalistic CAP setting where the participants were randomised either to intervention (ERST group) or to a control group that received the intervention at a later stage. Both groups received TAU during the study, which consisted of multidisciplinary CAP care.

### Procedure

**Fig. 1:**
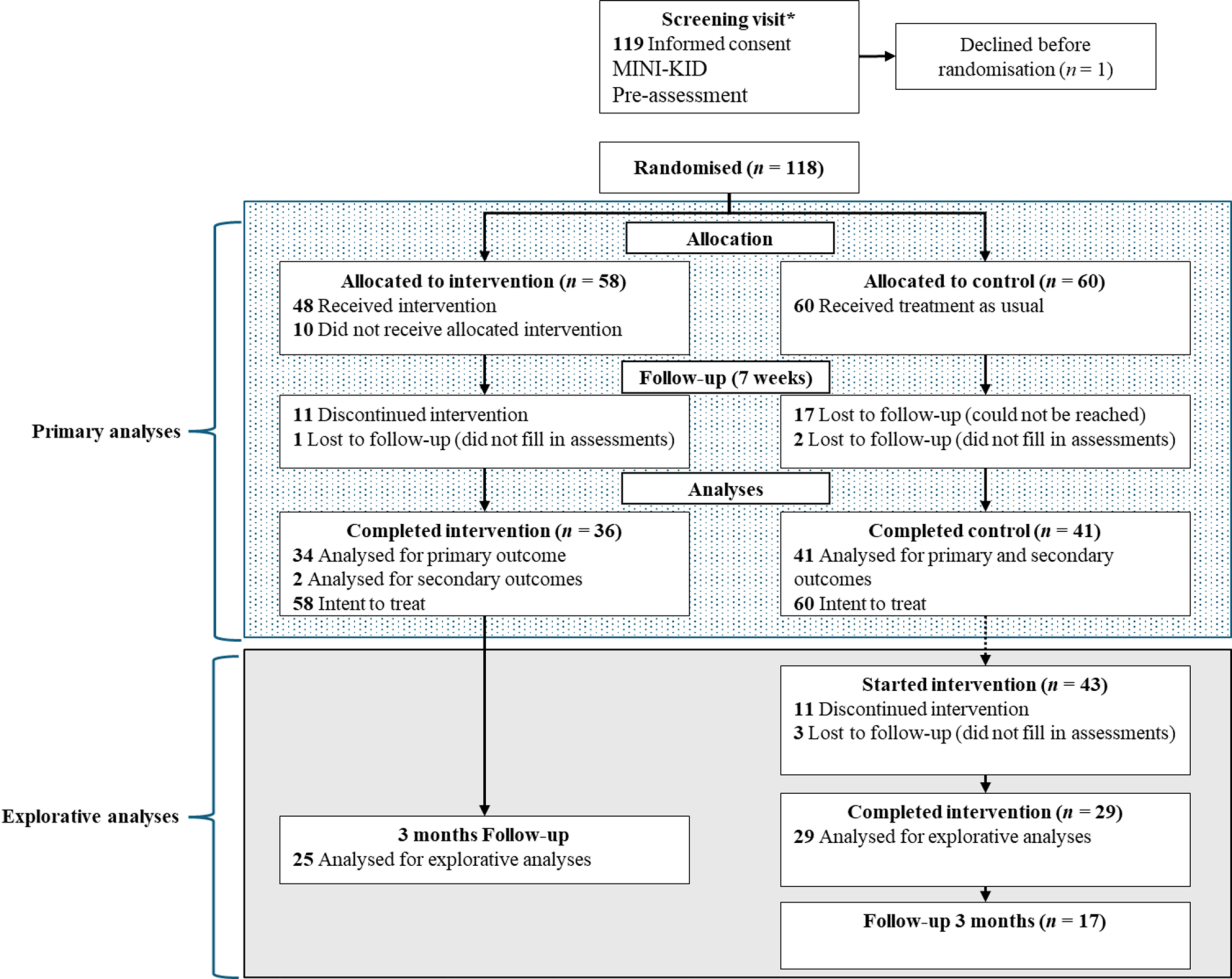
Participant flowchart. *Information on number of eligible participants who were approach by their clinician with initial study information and were not interested in further screening is lacking

Participants aged 14–17 years were recruited from two outpatient CAP clinics (Linköping and Norrköping) in the county of Östergötland, Sweden. Participants who presented with symptoms of ED were given written information about the study by their regular clinician and could register their interest to participate. Those interested were booked for a screening visit. However, no formal assessment of ED was conducted prior to inclusion. At the screening visit, additional oral and written information about the study was given, and participants and their legal guardians gave written informed consent. Participants were assessed for psychiatric symptomatology with the Mini-International Neuropsychiatric Interview for Children and Adolescents (MINI-KID) version 6.0 [39]. Those who did not meet any of the exclusion criteria and consented to participate were included and computerised randomised, ratio 1:1, to either ERST group (i.e., starting the intervention within two to three weeks after the screening visit) or to TAU control group (i.e., waiting to begin ERST group after approximately seven weeks). The main reason for this design was clinical feasibility. Recruitment was ongoing from March 2019 to January 2023. All participants filled in self-report measures at the screening visit, at the timepoint when the intervention group finished session 7, and again after three months. The control group started the intervention immediately after filling in post control measures and therefore the 3-months follow-up data could not be used for between-groups analysis, see Fig. 1. The treatment conditions were not concealed.

The trial was registered in Clinical Trials (ID NCT03900533) and was approved by the Regional Ethical Review Board of Linköping and the Swedish Ethical Review Authority (Dnr, 2015/264 − 31, 2018/512 − 32, 2019–05802, 2020–05670, 2022-00772-02, 2023-02435-02, 2024-02780-02).

### Participants

In total, 119 participants were included and 118 were randomised (See Fig. 1 for flowchart). The intervention was transdiagnostic and inclusion criteria were: being 14–17 years and enrolled at the CAP clinics in Norrköping and Linköping, Region Östergötland. Exclusion criteria were a diagnosis of schizophrenia, ongoing psychosis or mania, substance use disorder, severe anorexia and known/previously assessed intellectual disability. There was no formal assessment of intellectual functioning in the current study. Participants with high-functioning autism, autistic traits and/or borderline cognitive functioning were included if their level of functioning allowed it and if participation was feasible. Participants had at least one parent with them during the ERST group. The same parent participated in all sessions. There were no significant differences between groups in sociodemographic data and the primary outcome measure of ED measured with Difficulties in Emotion Regulation Scale (DERS; [5]) at baseline, see Table 1.

### Measures

Demographic information on gender, age, parental education, country of origin and living arrangements was collected. All measures in the current study were administered to the adolescents. ED was measured using the DERS [5]). DERS consists of 36 items measuring a general factor and six specific domains of ED; (a) Non-Acceptance of Emotions (Acceptance), (b) Inability to Engage in Goal-Directed Behaviours when Distressed (Goal), (c) Impulse Control Difficulties (Impulse), (d) Limited Access to Emotion Regulation Strategies (Strategies), (e) Lack of Emotional Awareness (Awareness), and (f) Lack of Emotional Clarity (Clarity). Scores range from 36 to 180 and higher scores indicate more ED. Cronbach’s alpha was α = 0.92 for the total scale, α = 0.86 for Acceptance, α = 0.86 for Goal, α = 0.91 for Impulse, α = 0.72 for Awareness, α = 0.86 for Strategies and α = 0.80 for Clarity in the present sample, indicating acceptable to excellent internal consistency. Alexithymia was measured with the Toronto Alexithymia Scale (TAS-20; [40, 41]). The questionnaire comprises three subscales: Difficulties identifying emotions (Identifying); Difficulties describing emotions (Describing) and Difficulties externalising emotions (External focus). Scores range from 20 to 100 and higher scores indicate higher levels of alexithymia. Internal consistency was α = 0.80 for the total scale, α = 0.80 for Identifying, α = 0.73 for describing and α = 0.58 for External Focus in this sample, indicating acceptable to good internal consistency, except for the External Focus subscale, which had a non-acceptable Cronbach’s alpha. This subscale’s internal consistency in the current sample is, however, in line with earlier studies [41, 42]. Symptoms of anxiety and depression were measured using Beck Anxiety Inventory (BAI; [43]) with scores ranging from 0 to 63 and Montgomery Åsberg Depression Rating Scale, self-report version (MADRS-S; [44]) ranging from 0 to 54. Higher scores indicate more symptoms for both measures. Cronbach’s alpha was α = 0.93 for BAI, and α = 0.89 for MADRS-S, indicating excellent and good internal consistency, respectively. Quality of life was measured with Brunnsviken Brief Quality of Life (BBQ; [45]), with good internal consistency in the current sample (α = 0.85). BBQ scores range from 0 to 96 and higher scores indicate higher quality of life. A shortened version of Levels of Emotional Awareness Scale for Children (LEAS-C; [6]) was used in addition to the self-reported measures. The original version consists of 12 described situations in which the child is asked to describe which emotions he/she (self) and another person (other) would experience in the described emotionally provoking situations. In the shortened version, four situations were selected that represent common emotions, such as fear, sadness and shame. Participants’ answers were rated by an external rater with experience of assessing LEAS-C. The rater was blind to participants’ treatment condition and whether answers were given before or after the intervention.

### Intervention

The ERST group included seven two-hour weekly sessions. Three months after the last session participants were given a booster session consisting of a summary of the content. The ERST group outlined here was influenced by third-wave, evidence-based cognitive behavioural therapy (CBT), focusing on facilitating behavioural change, mindfulness, and acceptance [46]. It places particular emphasis on emotional awareness, identifying and describing emotions, goal-directed behaviours and communication about emotions, primarily in the family context.

The conceptual model originates from acceptance-based behavioural therapies with ER identified as a core component. The main purpose was to provide psychoeducation about the nature and function of emotions, and at the same time provide skills to regulate emotions by increasing emotional awareness, identifying, describing, expressing and accepting emotions and engaging in goal-directed behaviours. The ERST group also included teaching participants skills to reduce judgment and vulnerability and to increase positive activities and validation (Table 2). Parents participated in group sessions together with the adolescents. Each session included a rehearsal of the previous session’s content and a run-through of the homework assignments. The actual content of the session, which was introduced by psychoeducation and illustrated with role play/video vignettes, was followed by exercises and discussions. More detailed information on the content and methodological aspects of the intervention is available in Holmqvist Larsson and Zetterqvist [47].

The ERST group was designed as an add-on treatment and both groups received TAU during the study. TAU consisted of multidisciplinary CAP care, such as pharmacological treatment and visits to nurses or counsellors. See supplement Table S1 for information on TAU.

The study involved 24 skills training groups (12 intervention groups and 12 control groups). The number of adolescent participants in the intervention groups ranged from two to six (M = 4.0, SD = 1.13). To be included in the final analyses, participants had to attend at least five sessions and fill in post assessment. Of the 36 adolescents in the intervention group included in the final analyses, 64% attended seven sessions, 25% six sessions and 11% attended five sessions. Drop-out rates were 38% in the intervention group and 32% in the control group. Drop-out for the control group meant that they could not be reached or did not complete post-control assessments. The most common reason (*n* = 10, 17%) for dropping out from the intervention group was that the session dates and times were inconvenient. These 10 participants did not begin the intervention. During the COVID pandemic some families were forced to drop out due to infections, restrictions or because the parents worked in healthcare and were not allowed to take time off. There was no statistically significant difference between the adolescents who did not come to the first session, dropped out or completed the intervention on the pre-intervention assessment on DERS (*F*(2,55) = 0.219, *p* = .54), TAS-20 (*F*(2,55) = 0.573, *p* = .57), BAI (*F*(2,55) = 0.188, *p* = .83) or MADRS (*F*(2,55) = 1.900, *p* = .16), as determined by one-way ANOVA.

In the intervention group, 11% (*n* = 4) adolescents had two parents participating jointly with them in the ERST group and 89% (*n* = 32) had one parent. When the control group were given the intervention, 7% (*n* = 2) had two parents participating and 93% (*n* = 27) had one parent.

### Skills trainers and supervision

Skills trainers were licensed psychologists, M.Sc. in psychology training to become licensed psychologist, counsellors, and licensed psychotherapist with employment in one of the two CAP-clinics. Psychologists in training worked together with senior skills trainers with extensive experience in ERST and CAP treatment. All skills trainers participated in a one-day training workshop conducted by the first author (KHL). The skills trainers received a comprehensive manual outlining the structure, content, and exercises for each session. Additionally, trainers were provided with supervision opportunities between sessions.

### Treatment adherence

To ensure adherence, sessions were audio recorded, and seven sessions were randomly selected for rating in accordance with an adherence checklist developed for the current study. The checklist included adherence to the content of each session, including, for example, emotional awareness, labelling emotions, the function of emotions, adherence to the method including items of going through homework, going through roleplays, following the structure of the session and adherence to the pedagogical approach, and the use of shaping, modelling and self-disclosure. Based on all the items in the checklist, the sessions were rated by the first author (KHL) and assessed as being adherent.

### Statistical analysis

Descriptive statistics were analysed using frequencies and percentages, mean values, and standard deviations, with cross-tabulation using chi square (χ^2^) and independent sample t-test for comparing differences between groups. For comparisons between pre-intervention measures for participants in the intervention condition that did not start, dropped out or completed the intervention, one-way analysis of variance (ANOVA) was executed. For between-groups analysis comparing the intervention to control group, one-way analysis of covariance (ANCOVA) was conducted with the pre-treatment value of the outcome variable used as covariate. The choice of ANCOVA as opposed to repeated measures ANOVA was based on its superior statistical power [48]. Treatment effects were evaluated for both completers and according to the intent-to-treat (ITT) principle. To enable the inclusion of all randomised participants in the ITT analyses, we used multiple imputation of missing data [49]. Specifically, we first ran a multiple regression analysis for each dependent variable at post-treatment, including pre-intervention measures of DERS, TAS-20, LEAS-C, BAI, MADRS-S and BBQ as predictors. Any variable significantly predicting post-treatment outcome was included as predictor in the multiple imputation model. Fifty samples were then imputed for each post-treatment measure using chained regressions. Finally, outcome was analysed in each imputed sample, and estimates pooled across samples. Statistical power was calculated using the website sealedenvelope.com for a continuous outcome superiority design, finding that with 118 participants, we would have 80% chance of detecting a true difference between groups representing a standardized mean difference of 0.52 at 5% significance level. Within-group analyses were executed using paired sample t-tests and one-way repeated measures. Effect sizes (Cohen’s *d*) were calculated using Cohen’s [50] criteria of 0.20, 0.50 and 0.80 for small, medium and large effects. For effect sizes in crosstabulation chi-square test, Cramer’s V was used, using Cohen’s criteria [50] of 0.10, 0.30, 0.50 for small, medium, and large effect. Internal consistency was calculated with Cronbach’s alpha. Statistical analyses were performed using the SPSS v. 29.0 software package (SPSS Inc, Chicago, IL) and StataCorp [51].

## Results

### Primary outcome

#### Emotion dysregulation

ITT analysis of ED measured with DERS showed a significant difference between the intervention group and the control group on the subscale Clarity, (difference = − 0.40, SD = 0.19; 95% CI − 0.78 to − 0.02; Cohen’s *d* = 0.65; *p* = .039), but not for the total scale or any other subscale of DERS, see Table 3.

Analyses were also conducted for the completers. After adjusting for pretest scores, there was no significant difference between the groups on DERS at post-assessment except for the subscale Clarity, *F* (1, 72) = 0.6.79, *p* = .011, Cohen’s *d* = 0.50 in line with results of the ITT analysis. For descriptive data on outcome measures, see Table 4.

### Secondary outcomes

#### Alexithymia and emotional awareness

ITT analysis was executed on TAS-20, measuring levels of Alexithymia, and a shortened version of LEAS-C, measuring emotional awareness, using multiple imputations. Results showed significant differences between intervention and control group on total TAS-20, (difference = − 0.34, SD 0.10; 95% CI − 0.55 to − 0.13; Cohen’s *d* = 0.61, *p* = .002) including subscales Identifying (difference = − 0.57, SD 0.17; 95% CI − 0.90 to − 0.23; *d* = 0.68; *p* = .001) and Describing (difference = − 0.64, SD = 0.18; 95% CI − 0.99 to − 0.29; *d* = 0.73; *p* = .001). There were no significant differences between the two groups on the shortened version of LEAS-C.

The analysis of completers confirmed the result from the ITT analysis with significant differences between the intervention group and the control group on TAS-20, *F* (1, 74) = 0.22, *p* = .002, *d*= 0.58, including the subscales Identifying and Describing, *F* (1, 74) = 13.57, *p* = < 0.001, *d* = 0.72 respectively *F* (1, 74) = 13.93, *p* = < 0.001, *d* = 0.71. There were no significant group differences on subscale External focus in TAS-20 or on the shortened version of LEAS-C. For pre and post mean values on secondary outcome measures for the intervention group and control group, see Table 4.

#### Psychiatric symptoms and quality of life

There were no significant differences between the intervention group and control group for symptoms of anxiety (BAI), depressive symptoms (MADRS-S) and quality of life (BBQ), see Table 3.

### Exploratory analyses

As statistical power tends to be higher for within-group designs [52], exploratory within-group analyses were also performed. Participants in the control group were offered the ERST group intervention immediately after completing the post control measures and could therefore be used as its own control. Paired sample t-tests were used to analyse the differences in pre and post scores (see Table S2 in supplement). Within the control group there were significant differences in DERS change scores between the control (*M* = 0.01, *SD* = 0.37) and intervention phases (*M* = 0.63, *SD* = 0.64), *t* (28) = − 4.04, *p* = < 0.001 (two-tailed). This was also true for all subscales; Non-acceptance [*t* (28) = − 2.30, *p* = .029 (two-tailed)], Impulse [*t* (28) = − 2.72, *p* = .011 (two-tailed)], Awareness [*t* (28) = − 3.38, *p* = .002 (two-tailed)], Strategies [*t* (28) = − 2.55, *p* = .017 (two-tailed)], Clarity [*t* (28) = − 3.60, *p* = .001 (two-tailed)], except Goals, see Table S2 in supplement. Alexithymia, measured by TAS-20, also showed significant differences in the total scale between the control (*M* = 0.03, *SD* = 0.34) and intervention phases (*M* = 0.46, *SD* = 0.57), *t* (26) = − 2.87, *p* = .004 (two-tailed) and for the Identifying subscale [*t* (26) = − 5.64, *p* = < 0.001 (two-tailed)]. Analyses showed no significant differences between mean scores of the shortened version of LEAS-C, psychiatric symptoms or quality of life in the different phases.

Three-month follow-up measures were completed by 74% of the participants in the intervention group who completed the primary outcome measure post-intervention. The participants in the control group received the intervention immediately after completing the post-control measures and it was therefore not possible to analyse their follow-up data. To explore the follow-up outcomes of the intervention, paired sample t-tests were conducted on pre-intervention to post-intervention, post-intervention to three-month follow-up data and for pre-intervention to follow-up for the participants in the intervention group. Analysis showed significant differences between pre- and post-intervention on ED measured with DERS on the subscales Goals [t (33) = 2.65, *p* = .012 (two-tailed)], Impulse [t (33) = 2.28, *p* = .030 (two-tailed)] and Clarity [*t* (33) = 2.15, *p* = .039 (two-tailed)], but not on the total scale (Table S3 in supplement). Levels of Alexithymia measured by TAS-20 showed significant reduction on the total scale [t (35) = 3.76, *p* = < 0.001 (two-tailed)] and on the subscale Describing [t (35) = 4.23, *p* = < 0.001 (two-tailed)]. The shortened version of LEAS-C showed significant increase in the subscale Self [*t* (32) = − 2.64, *p* = .013 (two-tailed)]. In post-intervention to 3-months follow-up analyses only the DERS subscales Impulse [*t* (23) = 2.09, *p* = .048 (two-tailed)] and Clarity [*t* (23) = 3.00, *p* = .007 (two-tailed)] showed significant changes. Pre-intervention to 3-months follow-up analyses showed significant changes on mean scores on DERS [*t* (24) = 3.22, *p* = .004 (two-tailed)], including the subscales Goals [*t* (24) = 2.52, *p* = .034 (two-tailed)], Impulse [*t* (24) = 3.49, *p* = .002 (two-tailed)], Awareness [t (24) = 3.05, *p* = .005 (two-tailed)] and Clarity [t (24) = 4.18, *p* = < 0.001 (two-tailed)]. Differences in mean scores on TAS-20 showed significant differences on the total scale [t (24) = 2.54, *p* = .018 (two-tailed)] and the subscales Describing [t (24) = 2.34, *p* = .028 (two-tailed)] and Identifying [t (24) = 2.11, *p* = .046 (two-tailed)] but not on the subscale External focus. Analyses showed no significant differences between mean scores of psychiatric symptoms or quality of life at the three timepoints.

## Discussion

In this randomised controlled trial, we examined the effect of a transdiagnostic and brief seven-session ERST group delivered jointly to adolescents and their parents in a naturalistic CAP setting. The ERST group significantly improved emotional clarity and levels of alexithymia compared to the active control group. There were no significant differences between the intervention group and control group concerning overall ED, psychiatric symptoms or quality of life. Results from the exploratory within-group analyses suggested a decrease in overall ED and indicate that there may be a further improvement emerging at three months post-intervention.

ANCOVA between-groups analyses showed significant differences in both ITT and completer samples, with a medium effect size (*ES* = 0.45 and *ES* = 0.50, respectively), between the intervention and control group on the DERS Clarity subscale. The intervention also resulted in significant reduction in alexithymia measured by TAS-20. Results showed a reduction in the total alexithymia scale, and in the subscales Identifying and Describing compared to the control group. The Clarity subscale of DERS conceptually corresponds to the Difficulties describing feelings facet of alexithymia [9], indicating that the results of these subscales should be consistent.

We hypothesised that the ERST group would result in reduced ED, reduced alexithymia, reduced symptoms of depression and anxiety and improved emotional awareness and quality of life. In conclusion, the between-group analyses only showed significant improvement in one of the subscales of the primary outcome measure, DERS. The secondary outcomes, alexithymia, symptoms of depression and anxiety, emotional awareness and quality of life, only showed significant changes for alexithymia measured with TAS-20.

In the analysis of the current ERST group, DERS was analysed using both total scale and subscales. The IERITA study [22], which consist of 12 weeks of internet-delivered ER treatment focusing on reducing NSSI, demonstrated overall ED improvements, which was not found in the current seven-session ERST group, although specific subscale data were not provided in IERITA.

Furthermore, earlier treatment research indicates that improvements in ED are associated with improvement in psychopathology [18, 53]. However, the analyses in the current study showed no significant changes in symptoms of anxiety or depression. These results are in line with prior randomised studies of brief interventions. IERITA showed improvement in self-reported ED but not in symptoms of depression or anxiety [22]. Similarly, a study of UP-A showed no significant reduction of depression or anxiety symptoms after eight weeks of treatment. However, after 16 weeks the intervention condition showed reduced symptoms of depression and anxiety compared to the waitlist (waitlist ran for 8 weeks) condition [27]. In one study of DBT-A, reduced symptoms of depression were found [28]. Noteworthy is that both length and intensity of this treatment differ significantly from the ERST group in the current study. In the study by Mehlum and colleagues, the DBT treatment consisted of 19 weeks of treatment, including weekly individual therapy and skills training group weekly, and also additional family therapy sessions and telephone coaching provided as necessary outside of scheduled therapy sessions [28]. ED was not specifically assessed in that study. In a meta-analysis exploring ER outcomes in psychological interventions for youth with depression and anxiety, mainly in high school and college samples, Daros et al. [25] stated that the relationship between improved anxiety and depression symptoms and improved engagement in ER skills was larger for studies with longer treatments. The findings of the present randomised controlled trial imply that only targeting ER in a brief intervention may not be sufficient for reducing general psychiatric symptoms in adolescents with more severe psychiatric conditions [22]. Given that patients in CAP clinics have moderate to severe symptomatology, high levels of comorbidities and low levels of functioning, a brief group intervention that only focuses on ER may not yield a significant impact on psychiatric symptoms over seven weeks.

Based on Gratz and Roemer’s conceptual framework of ER [5], the examined intervention emphasises increasing emotional awareness and clarity through identifying, describing, and labelling emotions. The first two sessions of the intervention focus on psychoeducation and practical exercises to improve these skills (see Table 2), which are then practiced throughout the following sessions. The results in the current study thus mirror the content of the intervention, in that the structure of the ERST group may be a contributing factor to the results on the Clarity subscale of DERS and the reduction of alexithymia.

An additional beneficial aspect of gaining knowledge and skills in how to identify, describe and communicate emotions is its potential influence on interpersonal relationships. A prior qualitative study of participants’ experience of the intervention [54] used in the current study, revealed that participants considered the improved parent-child relationship to be an important outcome and emphasised that improved emotional communication was a key component in this process. The participants reported that the skill of identifying and labelling emotions was a prerequisite for improved emotional communication between family members [54]. The potential benefit of including parents was not, however, assessed directly in the current study.

In contrast, the skills of engaging in goal-directed behaviours, acceptance and valued direction were introduced in the last sessions. Participants therefore had less opportunity to practice these skills, which might make them more challenging to generalise. In the exploratory analyses, which included follow-up data, a significant reduction in overall ED was observed in the intervention group at the booster session three months after the ERST group. Given the limited sample size for this analysis, and that analysis was explorative, these results should, however, be interpreted with caution.

Levels of emotional awareness measured with a shortened version of LEAS-C was chosen to complement self-reporting questionnaires. Participants’ responses were rated by an independent rater, blind to treatment condition and before and after status. Results of the shortened version of LEAS-C showed no significant results, either in the between-group analyses or in the explorative within-group analyses. This contrasts results from the feasibility study on the five-session ERST group, which found potential positive improvement on emotional awareness, measured with LEAS-C. To the best of our knowledge, LEAS-C has not previously been used as an outcome measure, and its sensitivity to detect changes may be limited. The analysis of the quality of life assessment showed no significant differences between the ERST group and the control group (TAU only) in the current study. However, UP-A and IERITA have previously shown improvements in functional level after ER interventions.

In DERS, emotional clarity and emotional differentiation are measured in the subscales Clarity and Awareness. These concepts are closely related to alexithymia, which is associated with various forms of psychopathology, including depression, anxiety disorders, risk behaviours, and NSSI [55]. Additionally, alexithymia is identified as a transdiagnostic risk factor for affective disorders [11]. As high levels of alexithymia hinder the selection of effective ER strategies, a focus on reducing alexithymia may be a way to increase the use of adaptive ER strategies [10]. Therefore, treatments focusing on improving ER skills can potentially be promising for disorders that frequently co-occur with alexithymia, such as autism and NSSI [56, 57]. There is evidence indicating that training focused on ER can lead to a reduction in levels of alexithymia [58, 59].

The within-group comparisons in the TAU control group with delayed ERST group, showed a significant difference between the intervention phase and control phase on total DERS including all subscales, except for Goals. Interestingly, there was an additional effect in the exploratory analyses in the three-month follow-up with reduced levels of ED after three months in total DERS scale, as well as in the subscales Goals, Impulse, Awareness and Clarity. The results should be interpreted with caution, however, due to a lack of dropout analyses, a small sample and that the analyses were explorative. However, there were no significant reductions in symptoms of depression and anxiety in the three-month follow-up.

The transdiagnostic CAP sample in the present study reported high baseline levels of ED compared to a community sample in the same age group [60]. Our results thus confirm earlier studies, which have reported similarly elevated levels of ED in clinical samples of adolescents [22, 61]. Even though there are currently no formal DERS cut-off scores for adolescents to compare with, it is interesting to note that in both these studies [22, 61], and in the present study, adolescents also reported high levels of ED post treatment even though dysregulation was reduced. Together these data support earlier research showing that clinical adolescent samples in general report high levels of ED [62] and further highlight the importance of targeting ER within these groups. In addition to high levels of ED, there was also substantial comorbidity in our sample, with more than a third of adolescents meeting criteria for three of more psychiatric diagnoses. The substantial overlap among diagnoses and high levels of comorbidity in clinical samples [63] are reasons for developing transdiagnostic treatment approaches that target core concepts of psychiatric conditions, such as ER, rather than focusing solely on a specific diagnostic set of symptoms.

### Strengths and limitations

This study was conducted in naturalistic CAP settings. This is a strength as the use of a clinical CAP transdiagnostic sample with few exclusion criteria in a clinical setting increases generalisability, which is typically a limitation in studies with more rigorous exclusion criteria. TAU is also an ecologically valid control. The study, however, has several limitations that must be addressed. Conducted within a naturalistic setting, the findings may be influenced by the context in which the study took place. Both the intervention group and the control group received TAU throughout the study which could potentially have affected the results. However, there were no significant differences between TAU in the intervention and control group. Thus, the naturalistic setting also presents drawbacks; notably, it limits the control over the sample population. The study also had some limitations due to the study design. Assessment was only collected pre, post and three months after ERST group. More data points during the intervention would have allowed for analysis of individual differences and trajectories of change. Assessment of outcomes could also have been strengthened if multiple informants were used, such as clinicians, preferably blinded to treatment group. In this study, both the total scale and the subscales of DERS were used. Earlier studies have used confirmatory factor analysis to examine if DERS measures a unidimensional or multidimensional phenomenon [64, 65]. Hallion et al. [64], for example, showed that a bifactor model excluding the Awareness subscale, with the addition of one general ED factor, resulted in the best fit. Monell et al. [65] confirmed the bifactor structure (excluding Awareness) and concluded that their findings provided further support for the validity of the previously established bifactor model. This indicates that DERS represents a multidimensional construct consisting of one general factor and other specific factors. Furthermore, it underscores the applicability of both the overall scale and its subscales, with the possible exception of the Awareness subscale. The external orientation subscale of TAS-20 had low Cronbach’s alpha in the current sample. This is in line with earlier studies on TAS-20 [40–42] and interpretations of this subscale therefore have to be made with caution. Similarly, the LEAS-C used here was a shortened version with unclear psychometric properties, which also indicates a need for cautious interpretation. In addition to the assessment of ER, alexithymia and symptoms in the current study, follow-up measurement of different impulsive or risk-taking behaviours associated with ED are lacking, as are outcome measures related to family functioning and the parent-child relationship, which could potentially have highlighted other treatment outcomes.

**Table 1:** Sociodemographic data, and DERS-score at baseline for Intervention (*N* = 58) sample and Control (*N* = 60) sample of adolescents

	Intervention *n* (%)	Control *n* (%)	Statistics	
			*p*	ES
*Gender*				
Boy	9 (15.5)	10 (16.7)	0.87	0.02
Girl	49 (84.5)	50 (83.3)	0.87	0.02
*Age (m*,* sd)*	15.55 (1.19)	15.75 (1.19)	0.37	0.17
*Parents’ education*				
Fathers with university/college education	17 (29.3)	23 (38.3)	0.26	0.11
Mothers with university/college education	26 (44.8)	29 (48.3)	0.85	0.02
*Country of origin*				
Adolescents born in Sweden	48 (82.8)	45 (75.0)	0.59	0.11
Fathers born in Sweden	45 (77.6)	42 (70.0)	0.20	0.18
Mothers born in Sweden	46 (79.3)	46 (76.6)	0.33	0.15
*Living situation*				
With both parents	31 (53.4)	38 (63.3)	0.33	0.09
Alternating between both parents	17 (29.3)	13 (21.7)	0.31	0.09
With one parent with or without new partner	7 (12.1)	6 (10.0)	0.70	0.04
Alone or with siblings or partner	1 (1.7)	1 (1.7)	0.97	0.00
In foster care or institution	1 (1.7)	2 (3.3)	0.59	0.05
Diagnosis*^,^				
ADHD/ADD	38 (65.5)	39 (65.0)	0.95	0.05
Depression	37 (63.8)	31 (51.7)	0.57	0.05
Anxiety disorder	32 (55.2)	27 (45.0)	0.27	0.10
Autism	9 (15.5)	12 (20.0)	0.52	0.06
PTSD	7 (12.1)	4 (6.7)	0.31	0.09
Eating disorder	5 (8.6)	4 (6.7)	0.69	0.04
ODD	3 (5.2)	4 (6.7)	0.73	0.03
OCD	5 (8.6)	1 (1.7)	0.09	0.16
3 or more diagnoses	23 (39.7)	19 (31.7)	0.37	0.08
*Suicidality*				
Suicidal thoughts	33 (56.9)	26 (43.4)	0.94	0.01
Suicide attempts	12 (20.7)	5 (8.3)	0.15	0.17
*DERS*				
Total scale	120.52 (21.07)	123.73 (23.04)	0.43	0.14
Nonacceptance of emotional responses	17.38 (5.85)	17.78 (6.46)	0.74	0.06
Difficulties engaging in goal-directed behaviour	21.33 (4.14)	21.38 (3.54)	0.94	0.01
Impulse control difficulties	20.41 (5.65)	21.33 (6.74)	0.41	0.15
Lack of emotional awareness	19.76 (4.58)	20.62 (4.77)	0.29	0.20
Limited access to emotion regulation strategies	25.91 (6.87)	26.57 (7.45)	0.80	0.05
Lack of emotional clarity	15.21 (4.39)	15.80 (4.48)	0.47	0.13

OCD = Obsessive compulsive disorder, ADHD/ADD = Attention Deficit Hyperactivity Disorder/Attention Deficit Disorder, PTSD = Post traumatic stress disorder. *Each participant could fulfil criteria for several diagnoses. Chi-square for group comparisons for categorical data and independent-sample t-test for comparisons for continuous data, effect sizes (Cramer’s V and Cohen’s d)

**Table 2:** Content of the emotion regulation skills training group

Sessions	Content
Session 1	Awareness of emotions and describing/labelling emotions
Session 2	Identifying emotions and the functions of emotions
Session 3	Primary and secondary emotions. Increasing validation and reducing judgment
Session 4	Reducing emotional vulnerability and imbalance, and increasing stability
Session 5	Making conscious choices – goal directed behaviours
Session 6	Acceptance and valued directions
Session 7	Summary, maintenance and generalisation of skills
Booster session	Overview and repetition

**Table 3:** ITT analysis and one-way between-groups analysis of covariance (ANCOVA). Pre and post mean scores on completers in control group and intervention group

	Intervention *n* = 33–36				Control *n* = 38–41				Completers analysis ANCOVA		ITT analysis *n* = 118		
	Pretreatment		Posttreatment		Pre control		Post control						
	*M*	*SD*	*M*	*SD*	*M*	*SD*	*M*	*SD*	*p*	*ES*	*95% CI*	*p*	*ES*
DERS total	3.38	0.49	3.13	0.73	3.40	0.66	3.35	0.74	0.13	0.32	− 0.44 to 0.08	0.17	0.30
Nonacceptance	2.84	0.99	2.71	0.98	2.93	1.10	2.94	1.11	0.36	0.17	− 0.63 to 0.22	0.34	0.20
Goals	4.40	0.67	4.01	0.91	4.26	0.72	4.10	0.97	0.20	0.28	− 0.54 to 0.26	0.48	0.18
Impulse	3.43	0.90	3.07	1.01	3.57	1.21	3.45	1.05	0.10	0.29	− 0.76 to 0.06	0.09	0.34
Awareness	3.40	0.65	3.10	0.85	3.33	0.76	3.34	0.90	0.08	0.37	− 0.58 to 0.11	0.17	0.30
Strategies	3.30	0.73	3.04	0.91	3.32	0.96	3.21	0.94	0.37	0.18	− 0.49 to 0.21	0.43	0.16
Clarity	3.08	0.87	2.73	0.85	3.09	0.92	3.18	0.89	0.01	0.50	− 0.78 to − 0.02	0.039	0.45
TAS-20 total	3.10	0.44	2.77	0.62	3.20	0.56	3.17	0.62	0.002	0.58	− 0.55 to − 0.13	0.002	0.61
Describing	3.46	0.66	2.85	0.92	3.40	0.93	3.43	0.90	< 0.001	0.71	− 1.0 to − 0.29	0.001	0.73
Identifying	3.11	0.66	2.80	0.76	3.29	0.86	3.49	0.84	< 0.001	0.72	− 0.90 to − 0.23	0.001	0.68
External focus	2.88	0.68	2.70	0.74	3.00	0.60	2.73	0.56	0.71	0.07	− 0.19 to 0.35	0.56	0.13
LEAS-C	3.36	0.66	3.52	0.45	3.32	0.46	3.32	0.65	0.18	0.29	− 0.10 to 0.41	0.22	0.26
Self	3.02	0.76	3.26	0.55	3.01	0.72	3.10	0.72	0.18	0.26	− 0.18 to 0.39	0.47	0.15
Other	2.81	0.76	3.03	0.55	2.76	0.67	2.89	0.62	0.37	0.18	− 0.09 to 0.50	0.17	0.29
BAI	1.37	0.59	1.31	0.59	1.17	0.64	1.19	0.67	0.79	0.04	− 0.25 to 0.17	0.69	0.06
MADRS-S	2.40	0.97	2.44	1.17	2.41	1.21	2.57	1.15	0.53	0.11	− 0.50 to 0.27	0.55	0.10
BBQ	7.21	3.69	7.59	3.80	7.88	3.63	7.42	3.26	0.38	0.03	− 0.65 to 2.12	0.29	0.03

DERS = Difficulties in Emotion Regulation Scale. Nonacceptance = Nonacceptance of Emotional Responses, Goals = Difficulties Engaging in Goal-Directed Behaviour, Impulse = Impulse Control Difficulties, Awareness = Lack of Emotional Awareness, Strategies = Limited Access to Emotion Regulation Strategies, Clarity = Lack of Emotional Clarity. TAS-20 = Toronto Alexithymia Scale. Describing = Difficulty Describing Feelings, Identifying = Difficulty Identifying Feeling, External focus = Externally-Oriented Thinking. LEAS-C = Levels of Emotional Awareness Scale for Children, shortened version. BAI = Becks Anxiety Inventory, MADRS-S = Montgomery Åsberg Depression Rating Scale, self-report version, BBQ = Brunnsviken Brief Quality of Life. ES = Effect size, Cohen’s d

**Table 4:** Descriptive data on outcome measures, pre, post and follow-up scores of intervention group and pre and post scores of control group

Measurement	Intervention		Control	
	Result	*n*	Result	*n*
*DERS total*				
Pretreatment	120.52 (21.07)	58	123.72 (23.04)	60
Posttreatment	109.85 (28.50)	34	120.07 (26.67)	41
3-month follow-up	102.10 (27.21)	21	–	–
*TAS-20*				
Pretreatment	61.55 (10.89)	58	65.00 (11.40)	60
Posttreatment	53.53 (14.79)	36	62.85 (11.21)	41
3-month follow-up	54.48 (12.81)	25	–	–
LEAS-C*				
Pretreatment	12.86 (2.69)	57	13.05 (2.29)	59
Posttreatment	14.03 (1.96)	34	13.28 (2.60)	39
3-month follow-up	14.13 (2.28)	23	–	–
*BAI*				
Pretreatment	28.88 (14.26)	58	25.83 (13.93)	60
Posttreatment	27.43 (12.33)	35	24.88 (13.87)	41
3-month follow-up	24.00 (13.20)	25	–	–
*MADRS-S*				
Pretreatment	22.79 (10.21)	58	21.97 (10.10)	60
Posttreatment	21.68 (10.63)	34	22.63 (10.88)	41
3-month follow-up	19.79 (12.02)	24	–	–
*BBQ*				
Pretreatment	41.09 (21.66)	58	44.43 (21.42)	60
Posttreatment	45.53 (22.78)	34	44.41 (19.65)	41
3-month follow-up	45.75 (19.87)	24	–	–

DERS = Difficulties in Emotion Regulation Scale, TAS-20 = Toronto Alexithymia Scale. LEAS-C = Levels of Emotional Awareness for Children. BAI = Becks Anxiety Inventory. MADRS-S = Montgomery Åsberg Depression Rating Scale, self-rating version. BBQ = Brunnsviken Brief Quality of Life Inventory. *Modified version

Furthermore, participants in the control group were offered the ERST group after the post-control assessment, which resulted in a lack of three-month follow-up data for between-group comparisons. There was also a gender imbalance in the sample, with a higher representation of girls, which may have skewed the results and limited their broader applicability. However, the proportion of females is typically larger in adolescent CAP samples [66]. Thus, further studies should attempt to achieve greater gender diversity among participants and include more participants and more data points with follow-up data for both groups. Due to the limited number of participants in the current study, a detailed examination of outcomes across different subgroups was not feasible. In the explorative analyses, multiple statistical comparisons were used, and therefore the increased risk of type I error must be considered when interpreting the results.

Finally, The COVID-19 pandemic had a significant impact on the study. The pandemic started during the initial phase of recruitment and was ongoing during almost the entire treatment phase of the study. It affected the delivery of the intervention and drop-outs. Skill trainers were required to wear face masks at various times and in a few cases sessions were delayed due to lockdowns. As a result of the pandemic, the number of participants who completed the ERST group was lower than anticipated.

### Conclusions and clinical implications

Results from this randomised controlled trial showed that a brief ERST group for adolescents and parents reduced participants’ difficulties in emotional clarity and improved the alexithymia facets of identifying and describing emotions compared to controls. Therefore, enhancing ER skills has potential in the treatment of adolescents in CAP settings. However, more comprehensive interventions might be needed to reduce more severe psychiatric symptoms.

## Supplementary Information

Below is the link to the electronic supplementary material.

Supplementary Material 1

## Data Availability

The datasets generated and/or analysed during the current study are not publicly available because we do not have participant or ethical approval to share data publicly. Data are available from the corresponding author upon reasonable request, [kristina.holmqvist.larsson@liu.se](mailto: kristina.holmqvist.larsson@liu.se).
