# Supplementary material for: Emotion regulation skills training for adolescents and parents in a clinical setting: a randomised controlled trial
Source: Child Adolesc Psychiatry Ment Health. 2026 May 28;20:85. doi: 10.1186/s13034-026-01098-1 (PMC13270657; doi:10.1186/s13034-026-01098-1)
Supplement: Supplementary file 1 — Supplementary Material 1 [file 13034_2026_1098_MOESM2_ESM.docx]

| **Table S1**  *Description of treatment as usual for completers, intervention group and control group* | | | | | |
| --- | --- | --- | --- | --- | --- |
|  | | Intervention  *n* = 28*  *n* (%) | Control  *n* = 25***  *n (%)* | Statistics | |
|  |  |  |  | *p* | *ES* |
| **Any ongoing psychopharmacological medication** | | 26 (92.9) | 21 (84.0) | .31 | .14 |
| **Type of medication**** | |  |  |  |  |
|  | Central stimulants | 15 (53.6) | 12 (48.0) | .39 | .12 |
|  | SSRI | 15 (53.6) | 10 (40.0) | .80 | .04 |
|  | Sleep medicine | 11 (39.3) | 12 (48.0) | .44 | .11 |
|  | Hydroxizin | 9 (32.1) | 6 (24.0) | .57 | .08 |
|  | Other | 1 (3.6) | 2 (8.0) | .46 | .10 |
|  |  |  |  |  |  |
| **Other treatment during intervention/control** | |  |  |  |  |
| **Physician** | |  |  |  |  |
| Number of visits | |  |  |  |  |
|  | 0 | 19 (67.9) | 17 (68.0) | .99 | .00 |
|  | 1 | 9 (32.1) | 5 (20.0) | .28 | .15 |
|  | 2 | 0 (0.0) | 2 (8.0) | .13 | .21 |
|  | 3 | 0 (0.0) | 1 (4.0) | .29 | .15 |
| **Nurse** | |  |  |  |  |
| Number of visits | |  |  |  |  |
|  | 0 | 22 (78.6) | 23 (92.0) | .17 | .19 |
|  | 1 | 5 (17.9) | 2 (8.0) | .29 | .15 |
|  | 2 | 1 (3.6) | 0 (0.0) | .34 | .13 |
| **Counsellor/Psychologist/Psychotherapist** | |  |  |  |  |
| Number of visits | |  |  |  |  |
|  | 0 | 13 (46.4) | 15 (60.0) | .32 | .14 |
|  | 1 | 5 (17.9) | 1 (4.0) | .11 | .22 |
|  | 2 | 6 (21.4) | 1 (4.0) | .06 | 26 |
|  | 3 | 1 (3.6) | 3 (12.0) | .25 | .16 |
|  | 4 | 0 (0.0) | 0 (0.0) | *na* | *na* |
|  | >5 | 3 (10.8) | 5 (20.0) | .35 | .13 |
| **Neuropsychiatric assessment** | |  |  |  |  |
| Number of visits | |  |  |  |  |
|  | 0 | 26 (92.9) | 22 (88.0) | .55 | .08 |
|  | 1 | 0 (0.0) | 0 (0.0) | *na* | *na* |
|  | 2 | 1 (3.6) | 2 (8.0) | .47 | .10 |
|  | 3 | 0 (0.0) | 0 (0.0) | *na* | *na* |
|  | 4 | 1 (3.6) | 1 (4.0) | .98 | .00 |
| **Other treatment**† | |  |  |  |  |
| Number of visits | |  |  |  |  |
|  | 0 | 24 (85.7) | 22 (88.0) | .92 | .01 |
|  | 1 | 2 (7.1) | 1 (4.0) | .62 | .07 |
|  | 2 | 0 (0.0) | 2 (8.0) | .13 | .21 |
|  | 3 | 1 (3.6) | 0 (0.0) | .34 | .13 |
|  | 4 | 1 (3.6) | 0 (0.0) | .34 | .13 |
| Note. *Missing data for six of intervention group and sixteen of controls, **Each participant could have several medications, †Other treatment: Physiotherapy, occupational therapy, psychiatric screening or school conference, *na* = not applicable, ES = Effect size. Chi-square for group comparisons and Cramer’s V for ES. | | | | | |

| **Table S2**  *Paired samples t-test comparing differences in DERS mean scores in the control group pre-control to post-control and pre-treatment to post-treatment (N = 21-29)* | | | | | | | | | | | | | | | |
| --- | --- | --- | --- | --- | --- | --- | --- | --- | --- | --- | --- | --- | --- | --- | --- |
|  | *N* | Pre-control | | Post-control | | Difference | Pre-treatment | | | Post-Treatment | | Difference | | *Statistics* | |
|  |  | *M* | *SD* | *M* | *SD* |  | | *M* | *SD* | *M* | *SD* |  | *p* | | *ES* |
| DERS (29) | 29 | 3.41 | .67 | 3.40 | .76 | .01 | | 3.40 | .76 | 2.77 | .58 | .63 | <.001 | | .75 |
| Nonacceptance | 29 | 2.95 | 1.11 | 3.03 | 1.14 | -.11 | | 3.03 | 1.14 | 2.53 | .95 | .50 | .03 | | .43 |
| Goal | 29 | 4.31 | .63 | 4.13 | .87 | .18 | | 4.13 | .87 | 3.60 | .82 | .53 | .19 | | .25 |
| Impulse | 29 | 3.43 | 1.23 | 3.45 | 1.11 | .18 | | 3.45 | 1.11 | 2.69 | .88 | .76 | .01 | | .50 |
| Awareness | 29 | 3.48 | .74 | 3.48 | .88 | -.01 | | 3.48 | .88 | 2.89 | .78 | .59 | .002 | | .63 |
| Strategies | 29 | 3.35 | .94 | 3.29 | .94 | .06 | | 3.29 | .94 | 2.64 | .87 | .66 | .02 | | .47 |
| Clarity | 29 | 3.12 | 1.04 | 3.17 | .95 | -.05 | | 3.17 | .95 | 2.37 | .67 | .79 | .001 | | .67 |
|  |  |  |  |  |  |  | |  |  |  |  |  |  | |  |
| TAS-20 (27) | 27 | 3.26 | .57 | 3.17 | .57 | .03 | | 3.17 | .57 | 2.81 | .59 | .46 | .008 | | .55 |
| Describing | 27 | 3.48 | .93 | 3.43 | .90 | -.05 | | 3.43 | .90 | 3.28 | .83 | .37 | .11 | | .32 |
| Identifying | 27 | 3.33 | .88 | 3.49 | .84 | -.25 | | 3.49 | .84 | 2.79 | .81 | .77 | <.001 | | 1.09 |
| External focus | 27 | 3.07 | .56 | 2.73 | .56 | .31 | | 2.73 | .56 | 2.54 | .58 | .25 | .72 | | .07 |
|  |  |  |  |  |  |  | |  |  |  |  |  |  | |  |
| LEAS-C | 24 | 3.47 | .43 | 3.27 | .77 | .20 | | 3.27 | .77 | 3.24 | .70 | .03 | .58 | | .12 |
| Self | 24 | 3.28 | .53 | 3.05 | .74 | .23 | | 3.05 | .74 | 3.14 | .62 | -.08 | .32 | | .21 |
| Other | 21 | 2.90 | .71 | 3.01 | .43 | -.12 | | 3.01 | .43 | 3.00 | .61 | .01 | .59 | | .12 |
|  |  |  |  |  |  |  | |  |  |  |  |  |  | |  |
| BAI | 25 | 1.23 | .66 | 1.27 | .70 | -.05 | | 1.27 | .70 | 1.23 | .64 | .04 | .57 | | .11 |
|  |  |  |  |  |  |  | |  |  |  |  |  |  | |  |
| MADRS-S | 27 | 2.61 | 1.12 | 2.66 | 1.20 | -.05 | | 2.66 | 1.20 | 2.40 | 1.18 | .26 | .26 | | .22 |
|  |  |  |  |  |  |  | |  |  |  |  |  |  | |  |
| BBQ | 24 | 7.21 | 3.82 | 7.01 | 3.09 | .19 | | 7.01 | 3.09 | 8.07 | 3.29 | -1.06 | .13 | | .32 |
|  |  |  |  |  |  |  | |  |  |  |  |  |  | |  |
| Note. DERS = Difficulties in Emotion Regulation Scale. Nonacceptance = Nonacceptance of Emotional Responses, Goals = Difficulties Engaging in Goal-Directed Behavior, Impulse = Impulse Control Difficulties, Awareness = Lack of Emotional Awareness, Strategies = Limited Access to Emotion Regulation Strategies, Clarity = Lack of Emotional Clarity. TAS-20 = Toronto Alexithymia Scale. Describing = Difficulty Describing Feelings, Identifying = Difficulty Identifying Feeling, External focus = Externally-Oriented Thinking. LEAS-C = Levels of Emotional Awareness Scale for Children, shortened version. ES = Effect size, Cohen’s d | | | | | | | | | | | | | | | |

| **Table S3**  *Paired sample t-test of mean scores from intervention group, pre, post and follow-up* | | | | | | | | | | | | | | | | |
| --- | --- | --- | --- | --- | --- | --- | --- | --- | --- | --- | --- | --- | --- | --- | --- | --- |
|  | Pre-Intervention | | Post-Intervention | | Follow-up | | | Pre-Intervention to Post-Intervention  (*n* = 33-36) | | | Post- Intervention to  Follow-up  (*n* = 23-25) | | | Pre-Intervention to  Follow-up  (*n* = 22-25) | |  |
|  | *M* | *SD* | *M* | *SD* | *M* | *SD* | *p* | | *ES* | *p* | | *ES* | *p* | | *ES* |  |
| DERS | 3.38 | .49 | 3.13 | .73 | 2.88 | .77 | .05 | | .35 | .08 | | .39 | .004 | | .64 |  |
| Nonacceptance | 2.84 | .99 | 2.71 | .98 | 2.47 | 1.07 | .41 | | .14 | .20 | | .27 | .15 | | .30 |  |
| Goals | 4.40 | .67 | 4.01 | .91 | 4.00 | .89 | .01 | | .45 | .37 | | .20 | .03 | | .45 |  |
| Impulse | 3.43 | .90 | 3.07 | 1.01 | 2.63 | 1.04 | .03 | | .39 | .05 | | .43 | .002 | | .70 |  |
| Awareness | 3.40 | .65 | 3.10 | .85 | 3.03 | .85 | .06 | | .34 | .10 | | .35 | .005 | | .61 |  |
| Strategies | 3.30 | .73 | 3.04 | .91 | 2.85 | 1.14 | .11 | | .29 | .40 | | .18 | .05 | | .41 |  |
| Clarity | 3.08 | .87 | 2.73 | .85 | 2.43 | .73 | .04 | | .37 | .007 | | .61 | <.001 | | .84 |  |
|  |  |  |  |  |  |  |  | |  |  | |  |  | |  |  |
| TAS-20 | 3.11 | .44 | 2.77 | .62 | 2.78 | .67 | <.001 | | .63 | .28 | | .22 | .02 | | .51 |  |
| Describing | 3.46 | .66 | 2.85 | .91 | 2.96 | .96 | <.001 | | .71 | .73 | | .07 | .03 | | .47 |  |
| Identifying | 3.11 | .66 | 2.80 | .76 | 2.73 | .86 | .07 | | .32 | .13 | | .31 | .05 | | .42 |  |
| External focus | 2.88 | .68 | 2.70 | .74 | 2.71 | .72 | .07 | | .32 | .75 | | .07 | .11 | | .34 |  |
|  |  |  |  |  |  |  |  | |  |  | |  |  | |  |  |
| LEAS-C | 3.41 | .73 | 3.52 | .48 | 3.50 | .56 | .10 | | .30 | 1.00 | | .00 | .53 | | .14 |  |
| Self | 3.09 | .67 | 3.23 | .56 | 3.31 | .44 | .01 | | .46 | .33 | | .21 | .12 | | .35 |  |
| Other | 2.84 | .87 | 2.96 | .62 | 3.10 | .59 | .12 | | .28 | .32 | | .21 | .14 | | .33 |  |
|  |  |  |  |  |  |  |  | |  |  | |  |  | |  |  |
| BAI | 1.37 | .59 | 1.31 | .59 | 1.15 | .63 | .41 | | .14 | .11 | | .33 | .12 | | .33 |  |
|  |  |  |  |  |  |  |  | |  |  | |  |  | |  |  |
| MADRS-S | 2.40 | .97 | 2.44 | 1.17 | 2.25 | 1.31 | .79 | | .05 | .09 | | .37 | .63 | | .10 |  |
|  |  |  |  |  |  |  |  | |  |  | |  |  | |  |  |
| BBQ | 7.21 | 3.69 | 7.59 | 3.80 | 7.63 | 3.31 | .54 | | .11 | .72 | | .08 | .68 | | .09 |  |
|  |  |  |  |  |  |  |  | |  |  | |  |  | |  |  |
| Note. DERS = Difficulties in Emotion Regulation Scale. Nonacceptance = Nonacceptance of Emotional Responses, Goals = Difficulties Engaging in Goal-Directed Behavior, Impulse = Impulse Control Difficulties, Awareness = Lack of Emotional Awareness, Strategies = Limited Access to Emotion Regulation Strategies, Clarity = Lack of Emotional Clarity. Follow-up = 3 months after finishing intervention. ES = Effect size, Cohen’s d. | | | | | | | | | | | | | | | | |
